# Higher Body-Mass Index and Lower Gray Matter Volumes in First Episode of Psychosis

**DOI:** 10.3389/fpsyt.2020.556759

**Published:** 2020-09-23

**Authors:** Marián Kolenič, Filip Španiel, Jaroslav Hlinka, Martin Matějka, Pavel Knytl, Antonín Šebela, Jiří Renka, Tomas Hajek

**Affiliations:** ^1^ Department of Applied Neuroscience and Neuroimaging, National Institute of Mental Health, Klecany, Czechia; ^2^ 3rd Faculty of Medicine, Charles University, Prague, Czechia; ^3^ Department of Complex Systems, Institute of Computer Science of the Czech Academy of Sciences, Prague, Czechia; ^4^ Department of Psychiatry, Dalhousie University, Halifax, NS, Canada

**Keywords:** obesity, schizophrenia, dyslipidemia, first-episode psychosis, low-grade inflammation, voxel-based morphometry

## Abstract

**Background:**

Neurostructural alterations are often reported in first episode of psychosis (FEP), but there is heterogeneity in the direction and location of findings between individual studies. The reasons for this heterogeneity remain unknown. Obesity is disproportionately frequent already early in the course of psychosis and is associated with smaller brain volumes. Thus, we hypothesized that obesity may contribute to brain changes in FEP.

**Method:**

We analyzed MRI scans from 120 participants with FEP and 114 healthy participants. In primary analyses, we performed voxel-based morphometry (VBM) with small volume corrections to regions associated with FEP or obesity in previous meta-analyses. In secondary analyses, we performed whole-brain VBM analyses.

**Results:**

In primary analyses, we found that when controlling for BMI, FEP had lower GM volume than healthy participants in a) left fronto-temporal region (pTFCE = 0.008) and b) left postcentral gyrus (pTFCE = 0.043). When controlling for FEP, BMI was associated with lower GM volume in left cerebellum (pTFCE < 0.001). In secondary analyses, we found that when controlling for BMI, FEP had lower GM volume than healthy participants in the a) cerebellum (pTFCE = 0.004), b) left frontal (pTFCE = 0.024), and c) right temporal cortex (pTFCE = 0.031). When controlling for FEP, BMI was associated with lower GM volume in cerebellum (pTFCE = 0.004). Levels of C-reactive protein, HDL and LDL-cholesterol correlated with obesity related neurostructural alterations.

**Conclusions:**

This study suggests that higher BMI, which is frequent in FEP, may contribute to cerebellar alterations in schizophrenia. As previous studies showed that obesity-related brain alterations may be reversible, our ﬁndings raise the possibility that improving the screening for and treatment of obesity and associated metabolic changes could preserve brain structure in FEP.

## Introduction

Neurostructural alterations are frequently reported already early in the course of schizophrenia (SZ) and may contribute to worse psychiatric outcomes (1, 2). Participants in their first episode of psychosis (FEP) typically show smaller gray matter (GM) volumes in frontal, temporal, and insular lobes, although there is heterogeneity in the direction and location of findings between individual studies (3–5). The reasons for this heterogeneity and the origins of neurostructural alterations in FEP remain unknown. They may represent neurodevelopmental antecedents but may also reflect the presence or absence of certain clinical factors (6, 7). Better understanding of the clinical factors which contribute to brain alterations in FEP is important for interpretation of findings. It is also the first step toward prevention or treatment of neurobiological changes, which may contribute to functional impairment in FEP (1, 2). One potential source of neuroimaging abnormalities in FEP could be the comorbidity with medical conditions known to affect the brain. One such condition is obesity.

Almost 1 in 2 participants with SZ are obese or overweight (40%–60%), which is significantly more than in the general population (8, 9). Overweight or obesity are disproportionately frequent already in the earliest stages of illness (10–12) and affect about 20% of participants with FEP (13). There is a highly replicated evidence showing that obesity is negatively associated with brain structure in non-psychiatric, otherwise healthy participants and already in adolescence (14, 15). The most pronounced effects of obesity are observed in frontal, mesiotemporal/limbic regions and cerebellum, brain areas which are also implicated in SZ (5, 16–21). Yet, we know little about the effect of obesity on brain structure in SZ. There is a single previous study, which documented additive effects of psychosis and overweight/obesity on a composite index of brain structure (BrainAGE) in participants with FEP (22). However, no studies have investigated the localization of obesity related neurostructural alterations in SZ and whether these directly overlap with regions associated with psychosis.

To address this knowledge gap, we studied the effects of both, obesity and psychosis on regional brain volumes in a large sample of individuals with FEP and healthy subjects. Our *a priori* hypotheses were that BMI will be negatively associated with brain structure, when controlling for FEP and individuals with FEP will have lower regional brain volumes relative to controls, when controlling for BMI. We also hypothesized that both BMI and FEP will be additively associated with brain structure in some regions of interest. In exploratory analyses, we further explored the links between clinical or obesity related biochemical alterations and the brain changes associated with FEP or BMI.

## Methods

### Sample Description

We analyzed a sample of 120 participants with FEP and 114 healthy participants (see **Table 1**). These analyses are a part of the Early Stages of SZ study (22, 23). To ensure generalizability, we recruited participants during their first hospitalization in a large general psychiatry hospital (1,200 beds), which serves the Prague and part of Central Bohemia regions—catchment area of over 1.5 million subjects. We focused on individuals with FEP, who met the following inclusion criteria: 1) were undergoing their first psychiatric hospitalization, 2) had the ICD-10 diagnosis of SZ (F20), or acute and transient psychotic disorders (F23) made by psychiatrist according to Mini-International Neuropsychiatric Interview (24), 3) had <24 months of untreated psychosis, and 4) were 18–35 years old. Participants with psychotic mood disorders were excluded from the study. As the diagnosis of SZ requires a minimal duration of symptoms, the retrospective diagnostic stability of SZ is low (0.6) (25). A significant number of individuals who are later diagnosed with SZ receive a different initial diagnosis. We wanted to recruit participants at the early stages of illness, to minimize the effects of illness and medications on brain structure. Thus, participants who were hospitalized before meeting the duration criteria for SZ are a particularly interesting group. These participants were included in the study and received the working diagnosis of acute and transient psychotic disorders, which is congruent with DSMIV brief psychotic disorder. This approach is in keeping with other studies of FEP (13, 25). Healthy participants, 18–35 years old, were recruited *via* advertisement, using the following exclusion criteria: 1) lifetime history of any psychiatric disorders, and 2) psychotic disorders in first or second-degree relatives. Additional exclusion criteria for both groups included history of neurological or cerebrovascular disorders and any MRI contraindications.

**Table 1:** Sample description.

	First-episode psychosis participants	Healthy participants	P
N	120	114	N/A
Sex, N (%) female	46 (38.33)	61 (53.51)	0.02
Age, mean (SD) years	27.00 (4.94)	25.70 (4.01)	0.03
Dg schizophrenia/acute polymorphic psychotic disorder N (%)	55(45.83)/65(54.17)	N/A	N/A
Duration of illness, mean (SD) months^a^	5.11 (5.43)	N/A	N/A
Duration of untreated illness, mean (SD) months^a^	3.12 (4.80)	N/A	N/A
Duration of antipsychotic treatment, mean (SD) months^a^	1.98 (2.92)	N/A	N/A
BMI, mean (SD; range)	23.32 (4.00; 16.0-42.5)	22.60 (2.93; 15.2–31.9)	NS
Overweight or obese, N (%)	38 (31.67)	23 (20.18)	0.045
LDL-cholesterol, mean (SD) mmol/l^b^	2.61 (0.73)	2.24 (0.56)	<0.001
HDL-cholesterol, mean (SD) mmol/l^b^	1.34 (0.37)	1.55 (0.38)	0.001
TG, mean (SD) mmol/l^b^	1.32 (0.56)	1.10 (0.45)	0.008
Total cholesterol, mean (SD) mmol/l^b^	4.56 (0.94)	4.29 (0.72)	0.05
CRP, mean (SD) mg/l^c^	2.16 (4.11)	1.18 (1.60)	NS
Glucose, mean (SD) mg/l^d^	4.45 (0.79)	N/A	N/A

BMI, body mass index; Dg, diagnosis; FEP, first-episode psychosis; HDL, high density lipoprotein; LDL, low density lipoprotein; TG, triglycerides; CRP, C-reactive protein.

^a^Data available from 116 FEP participants.

^b^Lipid levels were obtained in 73 FEP participants and 80 healthy participants.

^c^CRP levels were obtained in 68 FEP participants and 53 healthy participants.

^d^Three participants had glucose > 5.6 mmol/L, which in all instances normalized after re-testing; data available from 96 FEP participants.

Within 1 week from scanning, we collected information about, weight, height, blood pressure, duration of untreated/treated psychiatric illness, current medications, and personal history of hypertension by direct assessment verified by chart review. On the day of scanning, we obtained symptom ratings (The Positive and Negative Syndrome Scale—PANSS) and where available (in 73 FEP participants and 80 healthy participants), also fasting blood samples for biochemical analyses (total cholesterol, HDL-cholesterol, LDL-cholesterol and triglycerides). In participants who were medication naive prior to hospitalization (N = 40) we calculated cumulative medication exposure until MRI, based on their prospective inpatient charts. Additionally, for exploratory analyses, we were able to obtain blood levels of C-reactive protein in 68 FEP and 53 healthy participants. To exclude diabetes in FEP, we collected personal history verified by review of medical records (where available complemented by fasting glucose blood levels form the hospitalization records, 96 FEP participants). Biochemical analyses were performed in a single clinical laboratory using Siemens ADVIA 1800 Clinical Chemistry systems and standard clinical methods. We measured body mass index (BMI) using the formula: BMI = weight (kg)/height (meters)^2^. All diagnostic assessments and symptom ratings were performed by board certified psychiatrist using the Mini-International Neuropsychiatric Interview (24) and the PANSS (26).

The study was carried out in accordance with the latest version of the Declaration of Helsinki. The study design was reviewed and approved by the Research Ethics Board. Each participant received a complete description of the study and provided written informed consent.

### MRI Methods

All data were acquired on the same scanner using the same imaging sequences.

Specifically, we acquired T1-weighted 3D MPRAGE scans (TR = 2,300 ms, TE = 4.63 ms, FOV = 256 × 256 mm, bandwidth 130 Hz/pixel, matrix 256 × 256, voxel size 1×1×1mm3) on 3T Siemens Trio MRI scanner equipped with standard head coil.

### Voxel-Based Morphometry

We conducted FSL-VBM (27), http://fsl.fmrib.ox.ac.uk/fsl/fslwiki/FSLVBM), optimized VBM protocol (28) carried out with FSL tools (29). First, structural images were brain-extracted and gray matter-segmented before being registered to the MNI 152 standard space using non-linear registration (30). The resulting images (GM volume probability maps) were averaged and flipped along the x-axis to create a left-right symmetric, study-specific GM template. Second, all native GM images were non-linearly registered to this study-specific template and “modulated” to correct for local expansion (or contraction) due to the non-linear component of the spatial transformation. The modulated GM images were then smoothed with an isotropic Gaussian kernel with a sigma of 3 mm. Finally, voxel-wise general linear model (GLM) was used to compute associations between local GM volume as dependent variables, status - FEP versus healthy participants and BMI as predictors and age and sex as covariates of no interest; see *Statistical Analyses* for details). Permutation-based non-parametric testing, correcting for multiple comparisons across space, was applied. The threshold for primary analyses was set to p < 0.05 using threshold-free cluster enhancement (TFCE) and 5,000 permutations, as the cluster-based thresholding was developed to be more sensitive to finding true signal than voxelwise thresholding (31).

### Statistical Analyses

We used two approaches (primary and secondary analyses) to 1) test *a priori* hypotheses using small volume correction, but also to 2) perform exploratory, whole brain analyses in order to investigate associations between FEP or BMI and brain regions not included in the primary analyses. We consider these approaches complementary. Using only the small volume correction, we could miss signal in additional regions. Using only the whole brain analyses would enforce a purely exploratory approach when in fact there is enough evidence formulate *a priori* hypotheses. Our analytical plan included the following steps.

#### Primary VBM Analyses

These analyses were carried out on a combined sample of FEP (N = 120) and healthy participants (N = 114). In order to maximize the sensitivity to our studied conditions, we performed VBM analysis with small volume corrections to regions which have been previously associated with FEP or obesity. To do this, we created a mask, which combined the results of a spatial meta-analysis of voxel based morphometry studies, which investigated: 1) association between FEP and GM volumes (5), and 2) association between BMI and GM volumes (18), see **Figure 2S**. Using GLM, we assessed the associations between diagnostic status (FEP versus healthy participants) and BMI as explanatory variables and regional GM volumes as the dependent variable, while controlling for age and sex as covariates of no interest. We also tested for status × BMI interaction and included it in the model only if significant. We used BMI as continuous variable, as this is preferable for statistical reasons and to increase sensitivity. In addition, this was the preferred approach in most previous studies (18). As BMI is also used clinically to define categories with increased risk of adverse outcomes, based on validated cut-offs (32) we also repeated the analyses with BMI as a categorical variable. In these analyses we compared normal weight (BMI < 25) against overweight or obese participants (BMI >= 25). As our sample of FEP participants differed in age and sex compared to healthy participants, we also selected a subset of participants forming an age/sex-balanced dataset of 106 FEP and 107 healthy participants. We then performed sensitivity VBM analyses using the same approach as described above in this subsample.

#### Secondary Whole Brain VBM Analyses

These analyses were also carried out on a combined sample of FEP (N = 120) and healthy participants (N = 114). We conducted secondary whole brain VBM analysis and investigated the association between diagnostic status (FEP/healthy participants), BMI as explanatory variables, and local GM volumes, with sex and age as covariates of no interest. We tested for status × BMI interaction and included it in the model only if significant.

#### Additional Analyses

We explored the effects of clinical, treatment-related or metabolic variables on our VBM results among the FEP participants. Specifically, we tested for associations between average GM values from the voxels within the regions associated with FEP or BMI from the primary analyses and a) Duration of untreated psychosis; b) Duration of illness; c) PANSS positive subscale score; d) PANSS negative subscale score; e) PANSS global subscale score; f) Medication naive status before admission; g) Duration of antipsychotic treatment; h) chlorpromazine equivalent antipsychotic dose at MRI; i) cumulative medication exposure until MRI; j) Alcohol abuse history; k) THC history; l) Drug abuse diagnosis; m) Smoking at the time of MRI; n) HDL; o) LDL; p) TG; r) CRP; s) Glucose; t) Systolic blood pressure; u) Diastolic blood pressure; v) Age; and w) Sex. We used individual GM modulated, smoothed images (GM probability maps), and calculated average GM values from the significant clusters. For simple, robust and conservative exploration, we subsequently used Spearman’s correlation coefficient and Mann-Whitney U test to explore the association between average GM values and continuous or categorical variables respectively. We reported nominal p-values for these hypotheses generating/exploratory analyses.

## Results

We analyzed a sample of 120 participants with FEP and 114 healthy individuals (see **Table 1** for description of the sample).

### Primary VBM Analyses

**Figure 1:**
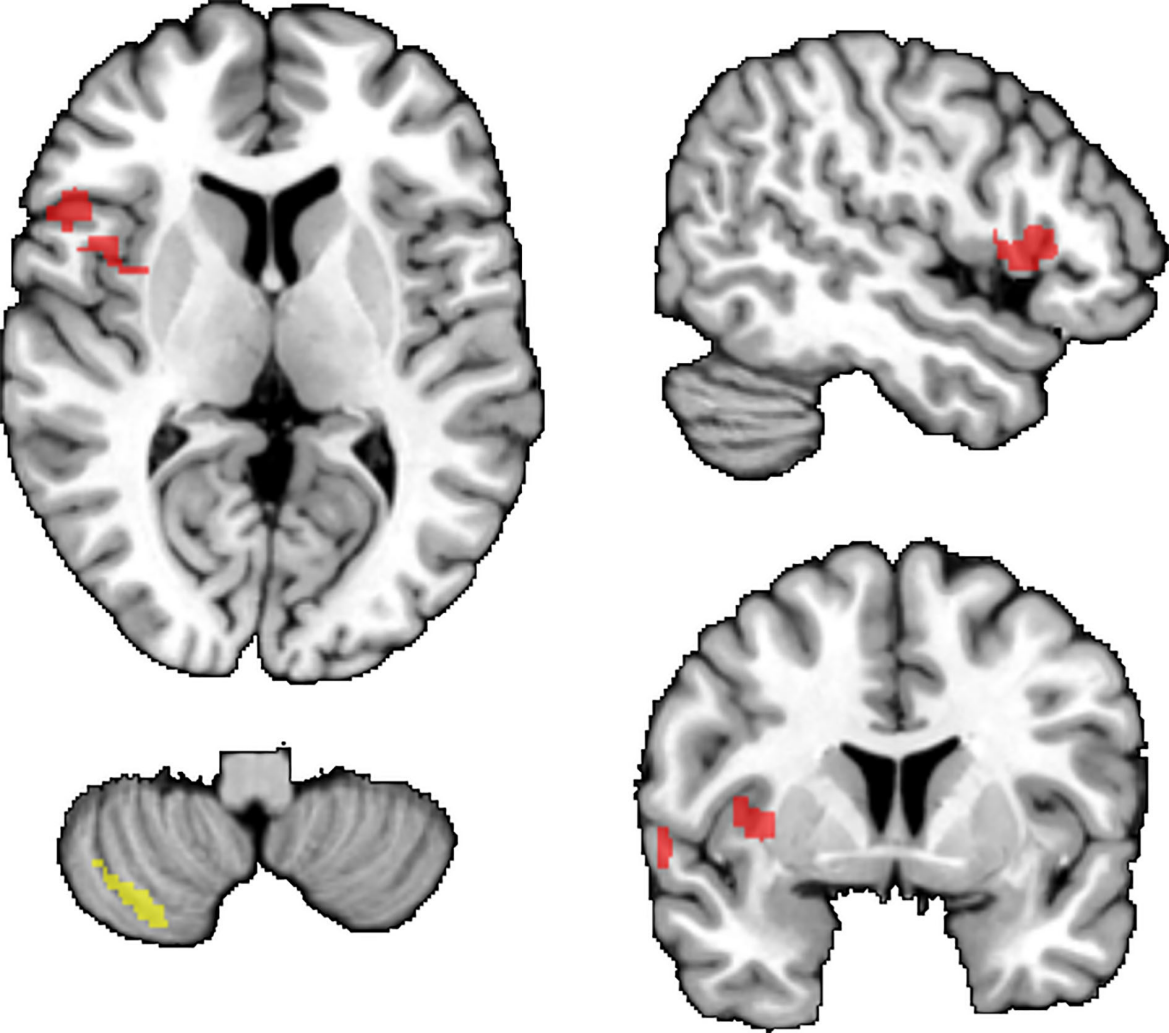
Results of primary VBM analyses (small volume correction). Brain regions where FEP had lower GM volumes than healthy participants (red). Negative associations between BMI and GM volumes (yellow). TFCE corr. p < 0.05. Results are displayed superimposed on the Colin 27 T1 template.

When focusing on regions previously associated with FEP or obesity, we found lower GM volume in FEP versus healthy participants, while controlling for BMI, in a) cluster including left IFG-STG-temporal pole-insula-operculum (Cohen’s d = 0.55; t_max_ = 4.19; pTFCE = 0.008; 395 voxels), b) left postcentral gyrus (Cohen’s d = 0.43; t_max_= 3.34; pTFCE = 0.043; 13 voxels). We also found a negative association between BMI and GM volume, when controlling for FEP, in the left cerebellum (Cohen’s d = 0.74; t_max_ = 5.30; pTFCE < 0.001, 144 voxels); see **Figure 1**. We did not find interaction between diagnostic status (FEP vs. healthy participants) and BMI. The results remained essentially unchanged, when using BMI as categorical predictor (normal weight vs. overweight/obese; see **Supplementary Material**). Sensitivity analyses on the age/sex-balanced dataset (N = 213) showed similar results as our primary analyses (see **Supplementary Material** and **Figure 1S**).

### Secondary Whole Brain VBM Analyses

**Figure 2:**
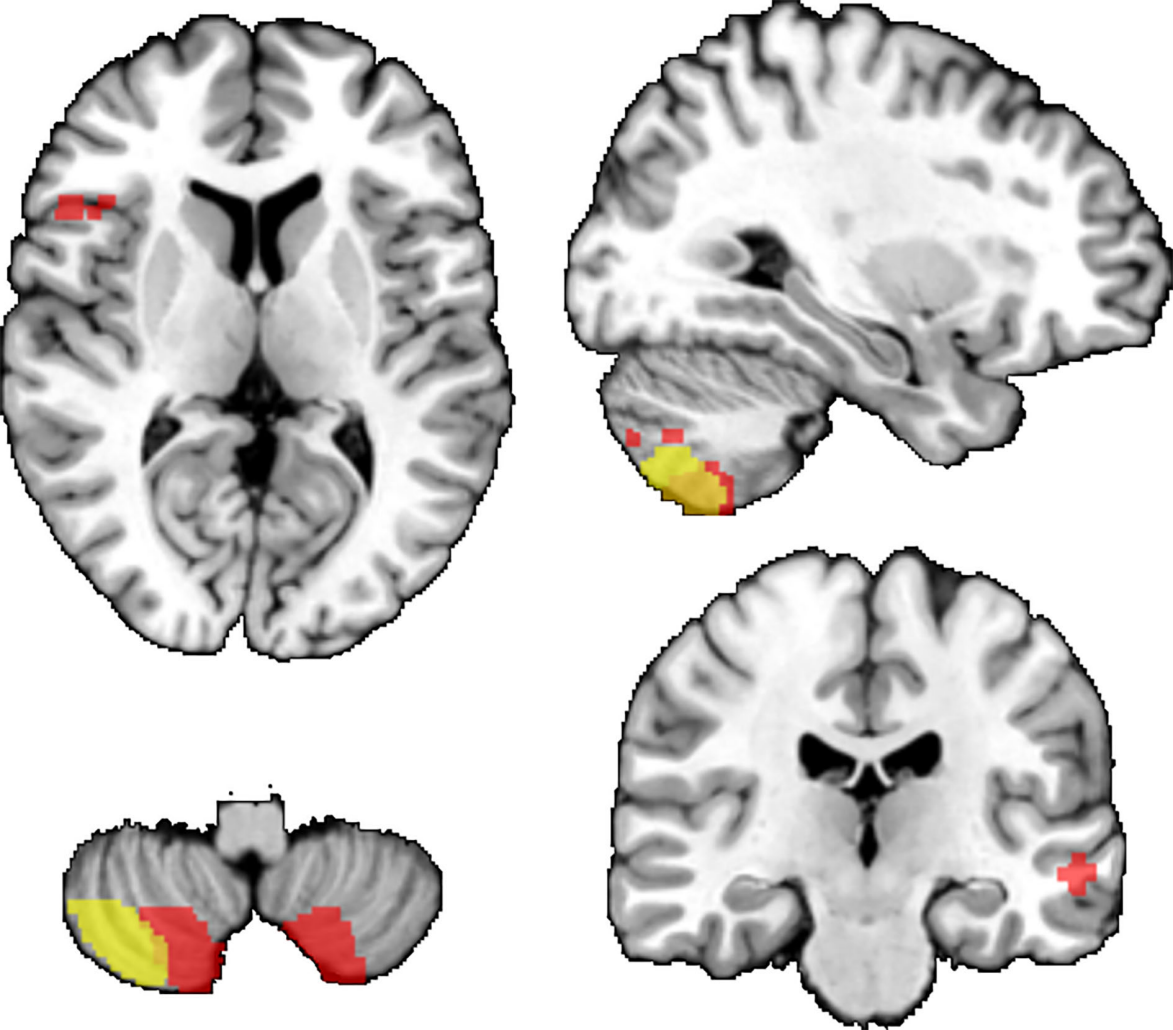
Results of secondary VBM analyses (whole-brain). Brain regions where FEP had lower GM volumes than healthy participants (red). Negative associations between BMI and GM volumes (yellow). Overlap between the associations with FEP and BMI (orange). TFCE corr. p < 0.05. Results are displayed superimposed on the Colin 27 T1 template.

At the whole brain level and when controlling for BMI, we found that FEP had lower GM volume than healthy participants in the a) right cerebellum (Cohen’s d = 0.57; t_max_ = 4.39; p_TFCE_ = 0.004; 1242 voxels), b) left cerebellum (Cohen’s d = 0.6, t_max_ = 4.59; pTFCE = 0.004, 1207 voxels), c) a cluster including left inferior frontal gyrus and superior temporal gyrus (Cohen’s d = 0.62; t_max_ = 4.7, pTFCE = 0.024, 151 voxels), d) right temporal cortex (Cohen’s d = 0.56; t_max_= 4.25; pTFCE = 0.031, 110 voxels). When controlling for FEP, BMI was negatively associated with GM volume in the cerebellum (Cohen’s d = 0.71; t_max_ = 5.1; p_TFCE_ = 0.004; 858 voxels); see **Figure 2**. We did not find interaction between diagnostic status (FEP vs. healthy participants) and BMI. Both FEP and BMI were negatively associated with GM in the left cerebellum, see **Figure 2**.

### Additional Analyses: GM and Clinical/Treatment-Related/Metabolic Variables in FEP Participants

Average GM values from voxels associated with BMI from primary analyses were negatively associated with low density lipoprotein cholesterol - LDL (r_s_ = −0.255, p = 0.030), high sensitive C-reactive protein - CRP (r_s_ = −0.327, p = 0.006), positively associated with high density lipoprotein cholesterol - HDL (r_s_ = 0.269, p = 0.021). None of the other clinical/treatment-related/metabolic variables were associated with average GM values from the voxels within the regions associated with BMI or FEP, see supplementary material and **Table 1S**.

## Discussion

In this study, we found that both FEP and BMI were negatively associated with local GM volumes. When controlling for BMI, individuals with FEP had lower GM volumes relative to controls in frontotemporal areas and right cerebellum. In addition, we found a partial effect of BMI when controlling for the diagnosis of FEP in the left cerebellum. There was also a cluster of lower GM volumes in the left cerebellum additively associated with both FEP and BMI. Our results are congruent with our *a priori* hypotheses and expand our previous study demonstrating effects of BMI on BrainAGE, a composite measure of brain structure (22).

Our findings are in keeping with previous studies in FEP or obesity. Lower GM volume in frontal and temporal brain regions are among the most replicated findings in VBM studies of FEP participants (3–5, 33). Recent meta-analysis using automated segmentation also confirmed the importance of these brain areas in FEP (34). The associations between BMI and cerebellar regions are also in line with previous studies and meta-analyses in general population (18). Interestingly, even our whole brain results fell into regions, which showed smaller volumes in obesity in previous meta-analyses (18), thus providing strong replication and increasing the chance of true positive findings.

Previous neurostructural findings regarding cerebellum in FEP are less consistent. In keeping with our results, a number of individual studies reported lower GM volumes of cerebellum in all stages of SZ (35–41), including those at ultra-high risk (UHR) for psychosis (39, 42), FEP (40), or in the largest such study, a multisite mega-analyses of 983 participants and 1,349 healthy controls (19). However, cerebellar findings from meta-analyses exploring GM volume abnormalities in FEP are inconsistent, with some reports finding cerebellar alterations only in certain subgroups or not at all (3–5). Previous studies did not control for BMI, which appears to be negatively associated with cerebellar volume independent of the effects of psychosis. Perhaps, the uncontrolled presence of overweight/obesity may contribute to heterogeneity in cerebellar volumetric findings between studies in FEP.

As obesity is a complex phenomenon, it is important to explore which obesity associated factors are particularly relevant to the brain alterations found in obese/overweight participants. These may include low-grade systemic inflammation (43–45), HPA dysfunctions (46, 47), leptin-resistance (48), insulin resistance (49–51), or ceramide-induced DNA methylation (52). Our findings provide the strongest support for the role of dyslipidemia and systemic inflammation in these brain alteration. Specifically, among FEP participants, LDL levels were negatively and HDL levels were positively associated with GM within the cluster showing association with BMI. This is the first report suggesting that lipid abnormalities could contribute to brain alterations in FEP. Additionally, CRP was negatively associated with average GM values from the cerebellar cluster associated with BMI (r_s_ = −0.327, p = 0.006). These findings are in line with previous studies suggesting a role of low grade chronic inflammation in obesity related brain alterations, especially in cerebellum (53–55).

We did not find associations between clinical/treatment-related variables, including antipsychotic treatment and GM volumes. This is surprising, as previous studies described associations between cumulative exposure of antipsychotic medication and GM volumes (56) as well as pro-adipogenic effect of antipsychotics (57, 58). But, at the same time, participants in our study had on average only 1.99 month of antipsychotic treatment and BMI was not associated with cumulative antipsychotic exposure. We cannot rule out that following a longer antipsychotic treatment, their metabolic side effects would become more relevant for association between antipsychotics and GM volumes. The class of antipsychotic medication could also play a role. Almost all of the participants in our study received second generation antipsychotics. Previous meta-analysis of longitudinal studies found GM volume reduction mostly in participants treated with first generation antipsychotics (59). However, other studies and meta-analyses have found negative effects of antipsychotics on brain structure even in studies, which included participants treated with atypicals (33, 60, 61).

The results of negative associations between BMI and GM volume in FEP are clinically concerning, as overweight/obesity is disproportionately frequent in psychoses (8, 13), as also conﬁrmed in this study. Identiﬁcation of higher BMI or overweight/obesity as a potential risk factor for neurostructural alterations in FEP may be the ﬁrst step toward their management. Lifestyle interventions focused on psychological well-being and weight management have proven to be effective in improving cognitive, clinical, and functional outcomes in many psychiatric syndromes (62–64). Obesity-related structural brain abnormalities might be reversible with dietary/lifestyle/surgical/medication interventions fostering weight loss and, especially in adolescents and young adults (65–69). Structural neuroimaging studies have reported increases in brain volumes following aerobic exercise in healthy subjects as well as in participants with SZ (70), which may be related to weight reduction (67, 71). Also medications targeting obesity may have neuroprotective effects. Promising results have been described using antidiabetic liraglutide (72), as also shown by our pilot trial in participants with bipolar disorder (73, 74). Other potential therapeutic options for future research include antiglucocorticoid mifepristone, which may improve cognitive dysfunction in participants with mood disorders (75) and showed also potential implications in reducing the risk of developing olanzapine/risperidone-induced weight gain (76, 77). Last but not least, protein deacetylase sirtuin1 (Sirt1) is protective against metabolic consequences of chronic exposure to a high-fat diet in animal models (78) and against signs of accelerated ageing in animal (79) as well as in human studies (80).

The results may have functional implications. Posterior cerebellar regions are connected to associative brain areas such as the prefrontal cortex and dysfunctions in the cortico-cerebellar-thalamo-cortical circuits could result in psychotic symptoms, as cerebellum acts as all-purpose modulator of movement as well as thought (81). Additionally, posterior cerebellum is involved in cognitive and mood regulation (82, 83). Therefore, cerebellar alterations could help explain the pathoplastic effects of obesity on psychiatric outcomes, as previously documented (84–87).

Besides the clinical/functional implications, our results are relevant for methodological reasons. BMI is not usually controlled for in VBM studies of FEP, although the negative effects of obesity on brain structure are robust and replicated in both psychiatric and non-psychiatric participants (17, 18, 88, 89). Identification of relevant contributors to GM abnormalities in FEP is an important step toward the better understanding and interpretation of neurostructural studies.

With regard to limitations, only a prospective study could establish the direction of the association between obesity and GM volumes. It is possible that obesity is not the cause, but rather the consequence of brain imaging changes, which may render participants more impulsive (90–92). We were unable to quantify the effects of chronic stressors, life events, diet, or exercise. Only our primary VBM analyses were testing *a priori* hypotheses using preselected variables and regions of interest. Additional analyses exploring associations between metabolic and inflammatory markers were exploratory/hypothesis generating and their results should be interpreted with caution. The fact that the healthy group did not match the patients is another limitation. At the same time, matching on unseen variables, which are only obtained as a part of the study is difficult and thus, similar previous studies also showed between group differences in metabolic markers (93). In addition, when we performed analyses on a subgroup of age and sex balanced participants (N = 213, 106 FEP, 107 healthy participants), the results remained essentially unchanged.

The study has several advantages, including the sample size (N = 234), focus on clinically interesting group of FEP participants and quantification of metabolic health using both, anthropometric as well as biochemical measurements. The results have good face validity, as they replicate some of the previous findings. The effects of metabolic alterations on brain health are of major interest in diabetology, yet remain markedly understudied in psychiatry, despite the high prevalence of metabolic alterations in this population.

To conclude, we demonstrated that higher BMI, overweight/obesity and diagnosis of FEP were significantly associated with lower regional GM volumes and that dyslipidemia and elevated CRP could contribute to obesity related neurostructural alterations. The effects of psychosis were most pronounced in frontotemporal regions, whereas both psychosis and BMI were additively associated with lower GM in the cerebellum. This is highly clinically relevant, as FEP participants have an increased risk of metabolic disorders and brain structural alterations. The additive effects of FEP and BMI also suggest that comorbidity with obesity could contribute to heterogeneity of neuroimaging findings in psychosis. It remains to be tested, whether treatment of overweight/obesity, dyslipidemia, and low grade systemic inflammation might improve or preserve brain structure in FEP and whether this would have positive impact on psychiatric prognosis.

## Data Availability Statement

The raw data supporting the conclusions of this article will be made available by the authors upon reasonable request and in compliance with the REB requirements.

## Ethics Statement

The studies involving human participants were reviewed and approved by Ethics committee of National Institute of Mental Health, Klecany, Czech Republic. The patients/participants provided their written informed consent to participate in this study.

## Author Contributions

All authors met ICMJE criteria for authorship. Specifically, MK, TH, FŠ, and JH contributed to the conception and design of the study. MK, TH, FŠ, PK, AŠ, JR, and MM contributed to data collection and preprocessing. MK, TH, and JH contributed to the statistical analyses and checking the accuracy and integrity of the data. MK, TH, and JH contributed to interpretation of the results. All authors contributed to the article and approved the submitted version.

## Funding

This study was supported by Ministry of Health of the Czech Republic (grant numbers 16–32791A and NU20-04-00393), by long-term strategic development financing of the Institute of Computer Science (RVO:67985807) of the Czech Academy of Sciences and by funding from the Canadian Institutes of Health Research (#142255). The sponsors of the study had no role in the design or conduct of this study; in the collection, management, analysis, and interpretation of the data; or in the preparation, review, or approval of the manuscript.

## Conflict of Interest

The authors declare that the research was conducted in the absence of any commercial or financial relationships that could be construed as a potential conflict of interest.
