# Supplementary material for: Higher Body-Mass Index and Lower Gray Matter Volumes in First Episode of Psychosis
Source: Front Psychiatry. 2020 Sep 23;11:556759. doi: 10.3389/fpsyt.2020.556759 (PMC7538831; doi:10.3389/fpsyt.2020.556759)
Supplement: Supplementary file 1 [file Table_1.doc]

**SUPPLEMENTARY MATERIALS.**

|  | | Average GM values - FEP cluster | Average GM value - BMI cluster |
| --- | --- | --- | --- |
| Age | rho | -,176 | -,031 |
|  | p | ,054 | ,736 |
| Sex | U | 1472 | 1897 |
|  | p | ,214 | ,293 |
| HDL | rho | -,145 | **,269*** |
|  | p | ,220 | **,021** |
| LDL | rho | -,139 | **-,255*** |
|  | p | ,241 | **,030** |
| TG | rho | ,211 | -,218 |
|  | p | ,073 | ,064 |
| CRP | rho | ,150 | **-,327*** |
|  | p | ,223 | **,006** |
| Alcohol abuse history | U | 78 | 16 |
|  | p | ,667 | ,291 |
| THC history | U | 1748 | 1724 |
|  | p | ,996 | ,894 |
| Drug abuse diagnosis | U | 985 | 1016 |
|  | p | ,759 | ,928 |
| Smoking at the time of MRI | U | 1633 | 1643 |
|  | p | ,964 | ,919 |
| Duration of untreated psychosis | rho | ,099 | -,130 |
|  | p | ,289 | ,165 |
| Duration of illness | rho | ,017 | -,028 |
|  | p | ,856 | ,768 |
| Glucose ^a^ | rho | ,000 | -,078 |
|  | p | ,999 | ,451 |
| Systolic blood pressure | rho | ,016 | -,093 |
|  | p | ,862 | ,324 |
| Diastolic blood pressure | rho | ,052 | ,018 |
|  | p | ,583 | ,847 |
| PANSS positive subscale score | rho | ,172 | -,024 |
|  | p | ,060 | ,797 |
| PANSS negative subscale score | rho | ,046 | ,054 |
|  | p | ,625 | ,567 |
| PANSS global subscale score | rho | ,115 | -,044 |
|  | p | ,225 | ,647 |
| Medication naive status before admission | U | 1237 | 1463 |
|  | p | ,181 | ,986 |
| Duration of antipsychotic treatment | rho | -,099 | ,097 |
|  | p | ,293 | ,302 |
| chlorpromazine equivalent antipsychotic dose at MRI | rho | -,073 | ,125 |
|  | p | ,446 | ,192 |
| cumulative medication exposure until MRI ^b^ | rho | ,036 | ,098 |
|  | p | ,810 | ,513 |

**Tab. 1S.**

Associations between average GM values from the primary analyses and clinical/treatment-related/metabolic variables in FEP participants.

We used Spearman's correlation (rho) and Mann-Whitney U test (U) as appropriate. We obtained lipid levels in 73 and CRP levels in 68 FEP participants.

* p<0.05

^a^ Glucose levels were available for 96 participants and were obtained from hospital records

^b^ Cumulative dose of antipsychotics was calculated only for 40 participants medication naive before admission

**VBM analysis using BMI categories****:**

(normal weight with BMI ≤25 vs. overweight/obesity with BMI >25)

When focusing on regions previously associated with FEP (Shah et al., 2017) or obesity (García-García et al., 2019), we found lower GM volume in FEP versus healthy participants, while controlling for BMI category, in a cluster including left IFG-STG-temporal pole-insula-operculum (t_max_=4.41; pTFCE=0.009; 379 voxels). We also found a negative association between overweight/obesity and GM volume, when controlling for FEP, in the left cerebellum (t_max_=5.39; pTFCE=0.002, 132 voxels). We did not found positive association between either FEP or overweight/obesity and GM volume.

**Sensitivity analyses:**

VBM analyses on age/sex–balanced subgroup of (N213) in the mask focusing on regions previously associated with FEP (Shah et al., 2017) or obesity (García-García et al., 2019), showed similar results as the primary analyses. We found lower GM volume in FEP versus healthy participants, while controlling for BMI, in a cluster including left IFG-STG-temporal pole-insula-operculum (t_max_=4.50; pTFCE=0.011; 429 voxels). We also found a negative association between BMI and GM volume, when controlling for FEP, in the left cerebellum (t_max_=5.56; pTFCE<0.001, 147 voxels) and in the right frontal medial cortex (t_max_=4.2; pTFCE=0.01, 61 voxels), see figure 1S.


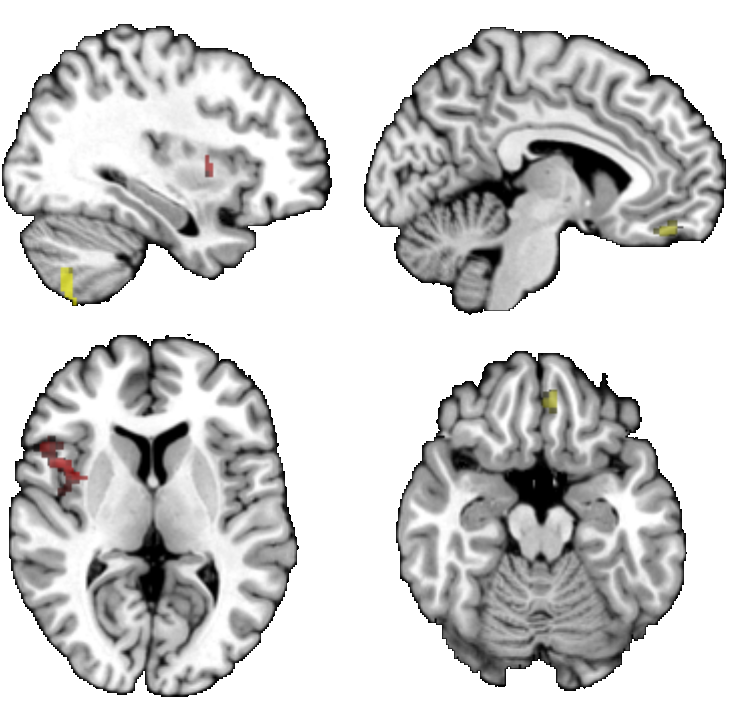


**Fig. 1S. – Sensitivity analyses results.**

Sensitivity VBM analyses on the age/sex-balanced subgroup of 106 FEP and 107 healthy participants. Lower GM volume in FEP versus healthy participants (red). Negative associations between BMI and GM volumes (yellow). TFCE corr. p<0.05. Results are displayed superimposed on the Colin 27 T1 template.


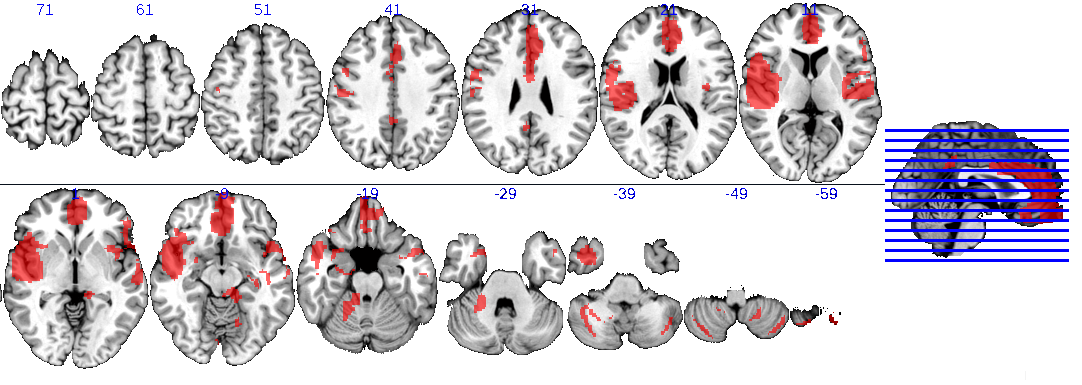


**Fig. 2S. - The mask for primary analyses**

The mask combined the results of a spatial meta-analysis of voxel based morphometry studies, which investigated: 1) association between FEP and GM volumes (5), and 2) association between BMI and GM volumes (30).
